# Severe Periodontal Disease Manifested in Chronic Disseminated Type of Langerhans Cell Histiocytosis in a 3-Year Old Child

**DOI:** 10.5005/jp-journals-10005-1269

**Published:** 2015-02-09

**Authors:** Monika Bansal, Vinay Kumar Srivastava, Rajesh Bansal, Vineeta Gupta, Manish Bansal, Shashikant Patne

**Affiliations:** Assistant Professor, Department of Dental Sciences, Institute of Medical Sciences, Banaras Hindu University, Varanasi, Uttar Pradesh India; Associate Professor, Department of Dental Sciences, Institute of Medical Sciences, Banaras Hindu University, Varanasi, Uttar Pradesh India; Reader, Department of Dental Sciences, Institute of Medical Sciences, Banaras Hindu University, Varanasi, Uttar Pradesh India; Associate Professor, Department of Pediatrics, Faculty of Modern Medicine, Institute of Medical Sciences, Banaras Hindu University, Varanasi, Uttar Pradesh, India; Assistant Professor, Department of Skin, Faculty of Modern Medicine, Institute of Medical Sciences, Banaras Hindu University, Varanasi, Uttar Pradesh, India; Assistant Professor, Department of Pathology, Faculty of Modern Medicine, Institute of Medical Sciences, Banaras Hindu University, Varanasi, Uttar Pradesh, India

**Keywords:** Floating teeth, Langerhans cell histiocytosis, Oste-olytic lesion, Seborrheic dermatitis.

## Abstract

Langerhans cell histiocytosis (LCH), previously known as histio-cytosis X, is a rare idiopathic disorder of reticulo-endothelial system with abnormal proliferation of bone marrow derived Langerhans cells along with a variable number of leukocytes, such as eosinophils, neutrophils, lymphocytes and plasma cells. Three years old male child presented with multifocal osteolytic lesions and papulosquamous skin lesions. Clinical and radio-graphic features, such as severe alveolar bone loss, mobility of teeth, precocious eruption of teeth, foating appearance of teeth in orthopantomogram (OPG), osteolytic lesion in skull and cutaneous lesions were highly suggestive of LCH disease. Skin biopsy confirmed a diagnosis of LCH. Induction chemotherapy with oral prednisolone and intravenous vinblastine was started. Child responded well to chemotherapy. The clinical significance of the presented case is to diagnose the case of LCH on the basis of the manifestation of severe periodontal disease as this can be first or only manifestation of LCH. A dentist plays a major role in the multidisciplinary treatment of LCH through routine examination and periodic follow-up.

**How to cite this article:** Bansal M, Srivastava VK, Bansal R, Gupta V, Bansal M, Patne S. Severe Periodontal Disease Manifested in Chronic Disseminated Type of Langerhans Cell Histiocytosis in a 3-Year Old Child. Int J Clin Pediatr Dent 2014;7(3):217-219.

## INTRODUCTION

Langerhans cell histiocytosis is a group of idiopathic disorders of reticuloen-dothelial system characterized by abnormal proliferation of bone marrow derived Langerhans cells.^1^ Abnormal proliferation of these cells replaces the bone and invades into the skin, mucosa and internal organs leading to tissue destruction. Langerhans cell histiocytosis was formerly known as histiocytosis X.^2^ The term histiocytosis denotes the proliferation of histio-cytes and other infammatory disorders and the letter X represents the unknown etiology of the disease. However, recently the terminology has changed to LCH or class I histiocytosis instead of histiocytosis X due to the fact that histiocytes are similar to the Langerhans cells present in the skin and mucosa.^3^ Langerhans cell histiocytosis is classified into three clinical forms depending upon the age and clinical presentation: (a) chronic localized form which includes unifocal or multifocal radiolucencies of bones and known as eosinophilic granuloma, (b) chronic disseminated form also known as Hand-Schuller-Chris-tian disease and (c) acute disseminated form also called as Letterer-Siwe disease.^4^ Langerhans cell histiocytosis can have an extremely variable presentation which can present difficulty in diagnosis. Our objective is to focus on the importance of changes in the periodontal tissues in a 3 years old male child having chronic disseminated type of LCH disease.

## CASE REPORT

**Fig. 1:**
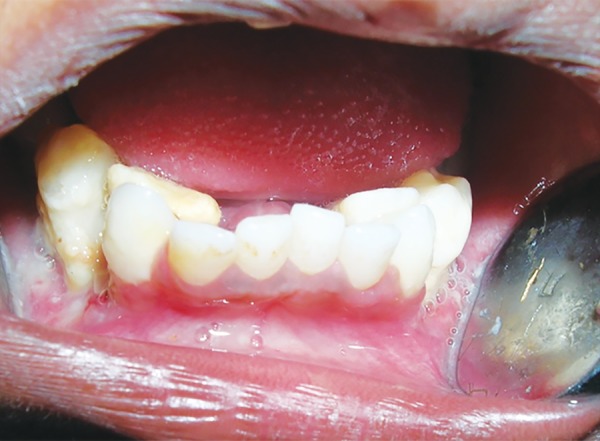
Intraoral examination showed generalized severe periodontitis

**Fig. 2:**
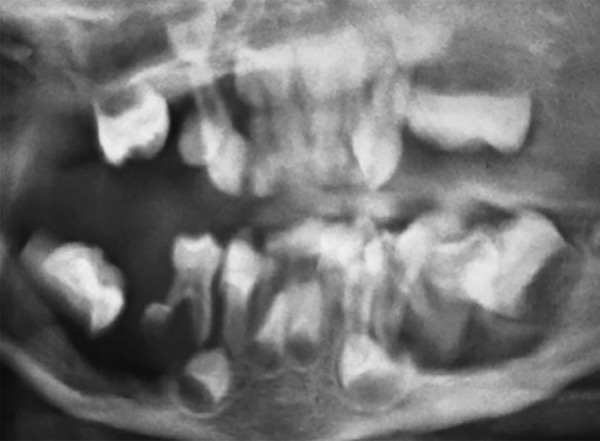
OPG X-ray revealed multiple radiolucent lesions and foating teeth

**Fig. 3:**
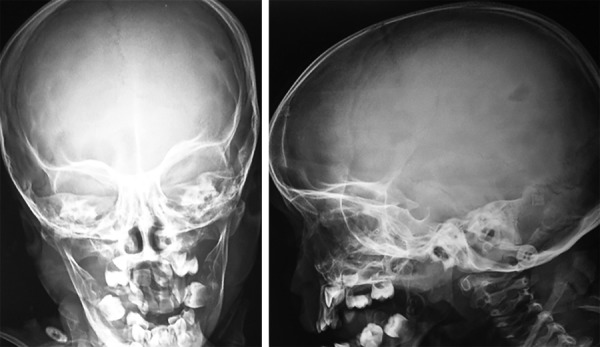
PA view of skull revealed the osteolytic lesion

**Fig. 4:**
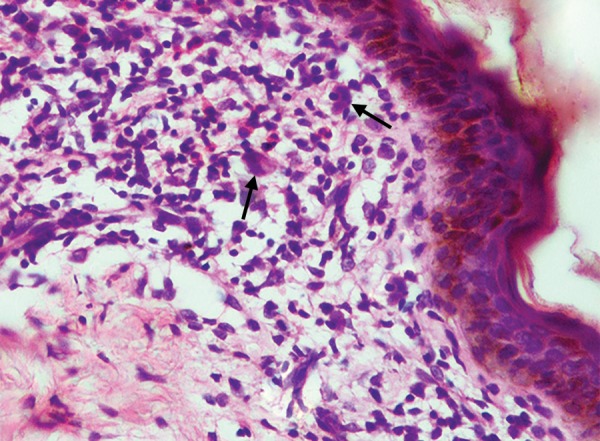
H&E staining at 400× showed Langerhans cells (arrows) and dense aggregates of infammatory cells in superficial dermis comprising of histiocytes, lymphocytes, eosinophils and plasma cells

A 3-year old male child reported at the Dental Outpatient Department, University Hospital, Varanasi, with the complaints of rapid loss of teeth for 7 months and difficulty in chewing food. On general examination, child was active with stable vitals and a short stature. A cervical lymph node was palpable. Liver and spleen were within normal range. Bilateral fine crepts were present on chest examination. Seborrheic dermatitis like papulosquamous lesions were present on the scalp, neck and shoulder region. Few hypopigmented macules were also present over the back and face. Nails of hand were deformed and showed atrophy. On intraoral examination, all deciduous teeth except maxillary right second molar, left first and second molar and precocious eruption of permanent first molars in all quadrants and both permanent mandibular lateral incisors were present. Poor oral hygiene, bleeding on probing and generalized severe periodontitis in the form of gingival recession was present (Fig. 1). All present deciduous posterior teeth as well as premature permanent teeth had grade III mobility. Complete blood count, thyroid profile, liver function test (LFT), Elisa for serotesting of human immunodeficiency virus (HIV), OPG X-ray, X-ray skull, X-ray chest and abdominal ultrasound were advised. Hemoglobin was 9.5 gm/dl with normal total and differential count. Thyroid function was within normal limits. LFT was normal except elevated alkaline phosphatase which was 1191 U/L. HIV test was nonreactive. OPG X-ray revealed multiple radiolucent lesions and foating teeth in the posterior region of maxilla and mandible due to severe alveolar bone loss and premature erupted permanent teeth do not have their roots (Fig. 2). X-ray of skull revealed multiple osteolytic lesions (Fig. 3). X-ray chest and abdominal ultrasound did not show any significant finding. After clinical, laboratory and radiographical examination, all features were suggestive of LCH and biopsy taken from one of the papulosquamous lesions present over the scalp was sent for histopathological examination. Histopathologic examination showed proliferation of langerhans cells and aggregates of infammatory cells comprising of histiocytes, lymphocytes, plasma cells and eosinophils (Fig. 4). Occasional langerhans cells showed numerous grooves and folds with abundant cytoplasm. T he sk in biopsy f nd i ngs were diag nost ic of LCH. I nduc-tion chemotherapy was started with weekly vinblastine and oral predisolone for 6 weeks followed by maintenance chemotherapy. Child showed good response and is in regular follow-up. Temporary prosthesis was also planned for replacement of missing teeth.

## DISCUSSION

The clinical presentation of LCH is variable and it mostly affects the skin and mucosa in the form of sebor-rheic dermatitis like lesions on face and scalp along with internal organ involvement. Involvement of teeth is an uncommon finding and rarely a presenting feature in most of the cases.

In the present case, clinical features of periodontal disease such as bleeding on probing, gingival recession, severe alveolar bone loss, and mobility of teeth in 3 years old child make the diagnosis difficult and it may be misdiagnosed as early onset periodontal disease. But other clinical and radiographic features such as precocious eruption of teeth, foating appearance of teeth in OPG X-ray, skull osteolytic lesion and skin lesions were suggestive of LCH disease. The oral manifestations may be the first sign of LCH disease and premature loss of deciduous teeth with bone loss and foating teeth is a clear sign of LCH disease.^5^ The present case was a case of multifocal single system due to presence of only bony lesions and skin lesions. In multifocal multisystem, LCH there is involvement of skeletal system, liver, spleen, hematopoietic system and lungs. Bony lesions were present in the X-ray of skull, maxilla and mandible and confirm the skeletal system involvement. Abdominal ultrasound, chest X-ray and complete blood count did not show any significant findings and excluded the involvement of liver, spleen, lung and bone marrow. A low Hb alone with normal TLC and platelet count can be because of iron deficiency anemia and will not classify as bone marrow involvement.

After clinical and radiographic findings, the LCH disease was confirmed on histopathologic examination of skin biopsy. The cell membrane of the histiocytes in LCH disease have CD1a antigen.^6^ Electron microscopy is also helpful in making the definitive diag nosis of LCH by identifying the specific marker known as ‘Birbeck granules’ in the cytoplasm of histiocytes. These granules possess tennis-racket morphology with transverse striations.^7^

Although the disease can present at any age but more than 50% of LCH cases are found in under the age of 10 years.^6^ LCH is most commonly found in males. The incidence of LCH disease ranges from 0.5 to 5.4 cases per million persons per year.^8^ The etiology and pathogenesis is still unknown. Various theories have been proposed regarding the possible etiology including immunologic reactions, viruses, bacteria or genetic involvement.^9-11^ Treatment of LCH includes surgery, radiotherapy, chemotherapy and steroid therapy alone or in combination depending upon the severity and extent of the disease.^12^ The present case was chronic disseminated type of LCH; therefore, chemotherapy was started including oral prednisolone and vinblastine intravenously. Patient has remained in regular follow-up.

The clinical significance of the presented case is that the severe periodontal disease may help in the diagnosis of LCH as these can be first or only manifestation of LCH and the dentist can play a major role in the multidiscip-linary treatment of LCH.
